# Factors Associated with Inadequate Management of Antiplatelet Agents in Perioperative Period of Non-Cardiac Surgeries

**DOI:** 10.5935/abc.20180162

**Published:** 2018-10

**Authors:** Juliana Maria Dantas Mendonça Borges, Pamella de Assis Almeida, Mariana Martins Gonzaga do Nascimento, José Augusto Soares Barreto Filho, Mario Borges Rosa, Antonio Carlos Sobral Sousa

**Affiliations:** 1 Universidade Federal de Sergipe, Aracaju, SE - Brasil; 2 Universidade Tiradentes, Aracaju, SE - Brasil; 3 Instituto Para Práticas Seguras no Uso de Medicamentos, Belo Horizonte, MG - Brasil; 4 Centro de Ensino e Pesquisa da Fundação São Lucas, Aracaju, SE - Brasil; 5 Departamento de Medicina da Universidade Federal de Sergipe (UFS), Aracaju, SE - Brasil

**Keywords:** Surgery/perioperative care, Intraoperative Care, Platelet Aggregation, Adults, Myocardial Infarction, Educational Status

## Abstract

**Background:**

The current guidelines dispose recommendations to manage antiplatelet agents in the perioperative period; however, the daily medical practices lack standardization.

**Objectives:**

To asses factors associated with inadequate management of antiplatelet agents in the perioperative period of non-cardiac surgeries.

**Methods:**

Cross-sectional Study conducted in hospital from October 2014 to October 2016. The study dependent variable was a therapy that did not comply with the recommendations in the Brazilian Association of Cardiology (SBC) guidelines. The independent variables included some characteristics, the people in charge of the management and causes of lack of adherence to those guidelines. Variables were included in the multivariate model. Analysis was based on the odds ratio (OR) value and its respective 95% confidence interval (CI) estimated by means of logistic regression with 5% significance level.

**Results:**

The sample was composed of adult patients submitted to non-cardiac surgeries and who would use acetylsalicylic acid (aspirin) or clopidogrel (n = 161). The management failed to comply with the recommendations in the guidelines in 80.75% of the sample. Surgeons had the highest number of noncomplying orientations (n = 63). After multivariate analysis it was observed that patients with a higher level of schooling (OR = 0.24; CI95% 0.07-0.78) and those with a previous episode of acute myocardial infarction (AMI) (OR = 0.18; CI95% 0.04-0.95) had a higher probability of using a therapy complying with the guidelines.

**Conclusion:**

Positive association between patients’ schooling level, or those with a history of previous AMI, with management of the use of aspirin and clopidogrel in the perioperative period of non-cardiac surgeries. However, diverging conducts stress the need of having internal protocol defined.

## Introduction

A study published in 20018 by the World Health Organization (WHO) informed that in 2012 313 million surgeries had been performed worldwide, thus evidencing a 38% increase in eight years. During that period in Brazil approximately 6 thousand surgeries per 100.000 inhabitants were performed, summing up about 10 to 13 million surgical procedures in 2012,^1^ and the rate of non-cardiac surgeries was estimated at 3 million per year.^2^ These figures still are bound to increase due to several factors, such as the growing and ageing population.^3^

In 2014, Botto et al.,^4^ stated that cardiac complications are the main cause of post-operation deaths of patients submitted to non-cardiac surgeries. These are alarming data once in the world over 10 million adults every year have at least one cardiac complication in the first 30 days following a non-cardiac surgical procedure.^4^^,^^5^ Among the cardiac complications arising from these types of procedure the most common is acute myocardial infarction (AMI),^4^^,^^6^^,^^7^ which is also associated with long-term mortality, although often enough it is detected earlier during clinical screening.^8^

Due to the key role performed by platelets in pathogenesis of atherothrombotic events, using antiplatelet agents is of the essence for primary and secondary prevention of cardiovascular events.^9^ However, although the use of antiplatelet agents has increased cardiovascular safety of many patients,^10^ when they need a non-cardiac surgery, surgeons and anesthesiologists frequently have to face the decision of whether to interrupt or not antiplatelet therapy in those patients during the perioperative period considering the risks of the occurrence of thrombi or bleedings, respectively.^11^^-^^13^

Thus, in order to help physicians make decisions in the perioperative period referring to antiplatelet therapy, the recommendations of the American association of thorax physicians (2012) and of the Brazilian (2013), European and American cardiology societies of cardiology (2014) are supposed to serve as basis of clinical evidence to help perioperative conducts and, consequently, to guarantee more safety to patients.^5^^,^^14^^-^^16^

In this sense, this study is an attempt to assess the factors associated with inadequate management of antiplatelet agents in the perioperative period of non-cardiac surgeries based on the existing Brazilian guidelines.

## Methods

### Study outline, sample and data collection

This is a cross-sectional study conducted in a high-complexity hospital, which is reference in cardiology and has internal hospital accreditation. That hospital unit contains 150 beds and, during the study period, 650 non-cardiac surgeries per month were performed on average.

In the study patients submitted to non-cardiac surgeries and who previously and regularly used at least one platelet agent for primary or secondary prevention were included, which composed a sample obtained by convenience instead of probabilistic, composed of adult patients (18 years old or older).

Data were collected from October 2014 to October 2016 by means of interviews with patients, or with their companions, before they were submitted to surgical procedures, using a questionnaire specific to obtain data. The interviews were held by a team of professionals and academics previously trained who attended the Departments of Pharmacy and Medicine of a public university and the Department of Pharmacy of a private university.

### Variables and data analysis

Descriptive analysis of the variables was done by determining absolute and relative frequencies for qualitative variables, and the means for quantitative variables. In the univariate and multivariate analyses the preoperative therapy with aspirin or clopidogrel was defined the dependent variable, which is inadequate according to the SBC recommendations (yes or no), once the study was conducted in Brazil. For this variable firstly was determined whether the patients had used antiplatelet agent for primary and secondary prevention, and then whether the recommendations disposed in the SBC guidelines referring to antiplatelet agents and anticoagulants in cardiology had been met, those adopted by the institution as reference at the time of the study, as presented in Box 1.

**Box 01:** SBC recommendation (2013) as to using aspirin and clopidogrel in the preoperative period of non-cardiac surgery

Indications	References
Patients using aspirin for secondary prevention in schedule of non-cardiac operations should keep using aspiring in a smaller dose (75 to 100 mg/day), except in neurosurgeries and transurethral resection of the prostate.	25,28
Patients using aspirin for primary prevention should suspend it 7 days before the procedure.	
For patients using clopidogrel as primary prevention, its use should be suspended 5 days before the surgical procedure.	26
For patients using clopidogrel for secondary prevention, the bleeding risk should be considered. When the bleeding risk is moderate or high, clopidogrel should be suspended 5 days before the procedure, but when the bleeding risk is low, the antiplatelet agent should be maintained.	29

ASPIRIN: acetylsalicylic acid.

Independent variables are described in Table 1. Patients were deemed to have a history of revascularization procedure if they had already been submitted to percutaneous coronary intervention or surgical revascularization. Patients were deemed dyslipidemic when they used medicines such as statins, resins, ezetimibe or fibrates, which the V Brazilian Guideline of Dyslipidemia and Atherosclerosis Prevention (2013) deems treatments of choice for dyslipidemia,^17^ additionally, patients were deemed hypertensive when at their medical records there was this information and because they used anti-hypertensive medicines, as described in the 7^th^ Brazilian Guideline of Arterial Hypertension (2016).^18^ For the Body Mass Index (BMI), patients who had 18.5- 24.9 Kg/m^2^ BMI^19^ were considered having normal weight. As to a surgery’ intrinsic risk of cardiac complications, the 3^rd^ SBC Guideline of Perioperative Cardiovascular Assessment^20^ was adopted as reference^20^.

**Table 1:** Sample characteristics (n = 161). High-complexity hospital, Aracaju, Sergipe, Brazil, 2014-2016

Characteristics	Total n(%)	Noncomplying with recommendations^*^		Value p^§^
		No(%)	Yes (%)	
**Gender**				
Male	73(45.3)	23.3	76.7	
Female	88(54.7)	15.9	84.1	0.237
**Age**				
40-69 years	85(52.8)	15.3	84.7	0.178
70-99 years	76(47.2)	23.7	76.3	
**Schooling**				
Up to high-school	65(40.4)	12.3	87.7	0.180
Primary School – complete – incomplete	40(24.8)	25.0	75.0	
University –complete or incomplete	56(34.8)	23.2	76.8	
**Married**				
No	63(39.1)	20.6	79.4	0.722
Yes	98(60.9)	18.4	81.6	
**Works**				
No	126(78.3)	23.0	77.0	0.022
Yes	35(21.7)	5.7	94.3	
**Has children**				
No	10(6.2)	10.0	90.0	0.443
Yes	151(93.8)	19.9	80.1	
**Body Mass Index^†^**				
Up to 29	115(71.3)	20.2	79.8	0.687
30 or more	46(28.7)	17.4	82.6	
**Number of diseases^‡^**				
0-2	120(74.5)	20.8	79.2	0.385
3-4	41(25.5)	14.6	85.4	
**Previous revascularization procedure^#^**				
No	124(77.0)	19.4	80.6	0.953
Yes	37(23.0)	18.9	81.1	
**Acute Myocardial Infarct**				
No	132(82.0)	15.9	84.1	0.022
Yes	29(18.0)	34.5	65.5	
**Stroke**				
No	152(94.4)	18.4	81.6	0.270
Yes	9(5.6)	33.3	66.7	
**Dyslipidemia**				
No	94(58.4)	25.5	74.5	0.017
Yes	67(41.6)	10.5	89.5	
**Systemic Arterial Hypertension**				
No	43(26.7)	16.3	83.7	0.563
Yes	118(73.3)	20.3	79.7	
Continuation				
**Time using aspirin or clopidogrel**				
1-4 years	72(44.7)	19.4	80.6	0.956
5 years or more	89(55.3)	19.1	80.9	
**Surgeon expertise**				
General or digestive system	67(41.6)	80.6	19.4	0.770
Urologist	11(6.8)	16.1	83.2	
Orthopedist	24(14.9)	83.3	16.7	
Other	59(36.7)	78.0	22.0	

ASPIRIN: acetylsalicylic acid; SBC: Brazilian Society of Cardiology;

^*^ Therapy according or noncomplying with the use of aspirin or clopidogrel therapy in the preoperative period, according to the SBC;

^†^ Body Mass Index = (weight in Kg) : (height in meters^2^);

^‡^ Number of diseases documented in the medical records and confirmed by patients on the date they were admitted for surgery;

^#^ history of percutaneous coronary intervention or surgical revascularization;

^§^ Obtained with Pearson chi-square test, significant when < 0.05.

We conducted univariate analyses using the Pearson chi-square test or Fisher exact test with expected frequency equal or lower than five. All variables were included in the multivariate model which, on its turn, was done with logistic regression. Multivariate analysis was based on the odds ratio (OR) value and its respective 95% confidence interval (CI^95%^), estimated by logistic regression. A 5% level of statistical significance was the criterion adopted to identify characteristics independently associated with the dependent variable. The likelihood-ratio test was used to compare the models, and the final models’ properness was assessed with the Hosmer-Lemeshow test. All statistical analyses were done with the Stata® statistic software package, version 12.

### Ethical aspects

This investigation was registered in the National Council of Ethics in Research - CONEP with the Certificate of Submission for Ethical Appreciation - CAAE no. 33899914.2.0000.5546, in compliance with the norms for scientific research involving human beings in Brazil. All individuals included in the study agreed to participate in the research by signing a free and informed consent (FIC).

## Results

Out of the total number of patients interviewed (n = 1,200), 161 were included in this study because they reported using at least one antiplatelet agent: Aspirin (156) and clopidogrel (5). Among those, 48 used antiplatelet agent for primary prevention (29.8%) and 113 for secondary prevention (70.2%). The patients were 69.5 years old on average (minimum = 42; maximum = 99; SD = ±10.5) and the majority was female (54.7%), in addition to having an average AMI of 27.8 kg/m^2^ (minimum = 17.3; maximum = 46.3; SD = ±5.5) and number of diseases 1.8 on average (minimum = 0; maximum = 4; SD = ±0.9). The majority had schooling up to high-school (40.4%) and the mean time of daily use of aspirin and/or Clopidogrel was 6.3 years (minimum = 1; maximum = 40; SD = ±6.8). Table 1 shows the characteristics of the sample in detail:

Out of the whole sample, 80.7% of the sample failed to comply with the SBC cardiology guidelines. As to types of noncomplying therapies, most of them occurred in cases where platelet agents was suspended as recommended, but within a longer period of time to that recommended in the guidelines, as detailed in Table 2.

**Table 2:** Results of noncompliance with the SBC recommendations for using aspirin and clopidogrel in preoperative periods of non-cardiac surgeries (n = 161) in high-complexity Hospital, Aracaju, Sergipe, Brazil, 2014-2016

Therapy^*^		Frequency n(%)
Compliance		31(19.3)
Non compliance	It was not suspend; it was supposed to be suspended	30(18.6)
	It was suspended; it was not supposed to be suspended	37(23.0)
	It was suspended; it was supposed to be suspended, but for a period longer than recommended	42(26.1)
	It was suspended; it was supposed to be suspended, but for a period lower than recommended	21(13.0)
Total		161(100)

ASPIRIN: acetylsalicylic acid; SBC: Brazilian Society of Cardiology;

^*^ Therapy according or noncomplying with the use recommended by the SBC for using aspirin or clopidogrel in preoperative periods according to the SBC.

As to the people in charge of rendering orientation for the management of antiplatelet agents, 85.1 % of the surgeons who rendered instructions to patients, in addition to 63.2% of the cardiologists, did it in disagreement with the recommendations in the SBC guidelines as to the use of aspirin or clopidogrel in the preoperative period of non-cardiac surgeries. As to the cardiac risks in surgical procedures to which the patients were submitted, according to the SBC^20^ guidelines for perioperative cardiovascular assessment, the majority (58%) of the procedures was classified as low cardiac risk (<1%), and none was classified high risk in this study.

Table 3 presents the results of multivariate analyses of the characteristics associated to the therapy lacking compliance with the recommendations for using aspirin or clopidogrel in preoperative period according to the SBC. After multiple adjustments, schooling up to university, complete or incomplete (OR 0.24; CI95% 0.7-0.78), and previous history of AMI (OR 0.18: CI95% 0.04-0.95) remained independently associated with the therapy lacking compliance with the SBC. .

**Table 3:** Results of the multivariate analysis of the characteristics associated with the therapy lacking compliance with the recommendations of use of aspirin or clopidogrel in preoperative periods according to SBC (n = 161) in high-complexity Hospital, Aracaju, Sergipe, Brazil, 2014-2016

Characteristic	OR (CI^95%^)^*^	Value p*^†^*
**Gender**		
Male	1.00	-
Female	2.22(0.74-6.68)	0.155
**Age**		
40-69 years	1.00	-
70-99 years	0.63(0.24-1.65)	0.354
**Schooling**		
Up to high school	1.00	-
Primary school complete or incomplete	0.46(0.13-1.66)	0.237
University complete incomplete	0.24(0.07-0.78)	0.018
**Married**		
No	1.00	-
Yes	1.28(0.47-3.48)	0.631
**Work outside the home**		
No	1.00	-
Yes	4.80(0.92-25.11)	0.063
**Has children**		
No	1.00	-
Yes	0.60(0.06-5.73)	0.655
**Body Mass Index^‡^**		
Up to 29	1.00	-
30 or more	1.24(0.43-3.54)	0.689
Number of diseases^§^	1.72(0.65-4.56)	0.279
**Previous revascularization procedure^#^**		
No	1.00	-
Yes	2.08(0.58-7.49)	0.261
**Acute Myocardial Infarction**		
No	1.00	-
Yes	0.18(0.04-0.95)	0.043
**Stroke**		
No	1.00	-
Yes	0.21(0.03-1.66)	0.138
**Dyslipidemia**		
No	1.00	-
Yes	1.00(0.24-4.17)	0.999
**Systemic Arterial Hypertension**		
No	1.00	-
Yes	0.22(0.04-1.27)	0.090
**Time using aspirin or clopidogrel**		
1-4 years	1.00	-
5 years or more	0.90(0.35-2.36)	0.837
**Surgeon expertise**		
General or digestive system	1.00	-
Urologist	3.30(0.33-33.09)	0.310
Orthopedist	1.38(0.32-5.88)	0.665
Other	0.75(0.28-2.03)	0.578

ASPIRIN: acetylsalicylic acid; SBC: Brazilian Society of Cardiology;

^*^ Odds Ratio (CI^95%^) estimated with the logistic regression method;

^†^ Logistic regression significant when < 0.05;

^‡^ Body Mass Index = (weight in Kg) : (height in meters)^2^;

^#^ History of Percutaneous Coronary Intervention or surgical revascularization;

^§^ Number of diseases documented in medical records and confirmed by patient on the date they were admitted for surgery – continuous variable.

## Discussion

The rather expressive frequency of therapies with aspirin and clopidogrel lacking compliance with the SBC guidelines’ recommendations (2013) in the perioperative period of non-cardiac surgeries was not observed in other studies once, as far as we are aware, this is the first one conducted in Brazil on this subject. However, the lack of organization of standardization of medical conducts in the management of antiplatelet agents is well known, i.e., there are groups of physicians who advocate the suspension of those medicines before surgeries in order to avoid bleedings, while others advocate their maintenance in order to avoid thrombotic events.^11^^-^^13^^,^^21^^-^^24^

The Brazilian guidelines say that in cases where aspirin or clopidogrel is used for primary prevention of cardiovascular diseases, they should be suspended, respectively seven and five days before a non-cardiac surgical procedure. However, in this study, the majority of the noncompliance with the Brazilian guidelines happened due to their suspension for periods longer than those disposed for aspirin and clopidogrel. This conduct can potentially expose patients to cardiac complications in the perioperative period once the literature evidences that those medicines, after being suspended for 8-10 days, lose their antiplatelet agent’s effect.^25^^,^^26^ Cases where the conduct of suspending the drug was correct were also observed, but for a period shorter than that recommended in the guidelines and, so, the goal of losing the pharmacological effect of the antiplatelet agent is never reached once that effect at platelet level is irreversible, and the time they remain active is approximately 10 days.^26^

Therefore, although the conduct of suspending the antiplatelet agent was correct, it is possible to infer that the suspension of the antiplatelet agent for longer or shorter periods than those recommended by the guidelines occurred because the hospital does not have its own assistance protocols focused on this matter, and, as such, diverging conducts strengthen the need of defining internal conducts, more divulgation of the guidelines used as reference at that institution, and continued education. Double checking conducts according to internal protocols of an institution can also be an important choice to ensure patients’ safety.

Other important datum in this study, and one that draws attention, is that a significant number of noncomplying therapies occurred resulting from having patients oriented to suspend antiplatelet agents when the Brazilian guidelines state the opposite for cases where patients use aspirin and clopidogrel for secondary prevention of cardiovascular diseases,^24^^,^^27^ except for clopidogrel, which depends of the procedure’s bleeding risk;^28^ but in this case, all 5 patients who had been using this drug were submitted to low bleeding-risk surgeries. According to some authors, an increased bleeding risk related to the effect of the antiplatelet action of those drugs is well known,^29^^,^^30^ mainly in the ageing population,^31^ which stands for the majority in this study.

However, other studies, as much as the SBC orientations (2013), except for neurosurgeries and transurethral resection of the prostate, advocate that the benefits of secondary prevention substantially exceeds the bleeding risks those drugs may cause^13^^,^^24^^,^^27^ once the AMI is the main cause of death in old patients after non-cardiac surgeries.^32^

A successful surgery depends on the aptitude and technical skills of the surgeon, on the indication and previous preparation, on the perioperative period management and care dimensioning the risks, on preventing and treating complications.^33^ In other words, a surgeon operates trying to avoid surgical complications during the procedure as much as possible, and among them one can be highlighted among general complications, whose universal example is hemorrhage.^34^ Those statements can justify the results of this study because the medical expertise representing the majority of the results noncomplying with the guidelines was surgery.

As to the association with patients’ characteristics, it was observed that patients with more schooling and those who at some moment had an AMI episode have more chance of using antiplatelet therapy in the preoperative period of non-cardiac surgeries according to the SBC (2013). No studies with this type of association were found in the literature.

However, on this matter, in a research done in the United States, its findings strongly suggest that the level of schooling is able to affect the risk of an individual developing cardiovascular diseases, regardless of any cardiovascular risk factor defined, i.e., patients with less than 12-year schooling ran significantly higher risk of AMI than those with 12-year or more schooling.^35^ As much as other authors, we understand that a higher schooling level enables patients to understand better the doctor’s orientations as to managing medicines and their health condition, as much as to have more access to information,^36^ once nowadays patients would rather participate more and more in the decision-making process with their doctors.^37^

As to patients who already had an AMI episode and are in the group where the antiplatelet therapy complies more with the guidelines in the perioperative period, one can understand that surgeons and doctors in charge of this medicine management look for avoiding reinfarction, and so they instruct their patients not to suspend aspirin or clopidogrel in the preoperative period of non-cardiac surgeries, thus abiding by the recommendations in the guidelines and advocated by other authors.^15^^,^^24^^,^^25^^,^^27^^,^^38^

This study has some limitations once the information obtained about management of antiplatelet therapy was rendered by the very patients, or by their companions, who in some situations said that opinions diverged between surgeon and cardiologist, or between surgeon and anesthetist, for instance, which would lead the very patients, or their companions, to decide which orientations should be followed. Additionally, the answers were written down on the patients’ reports, and physicians did not have the opportunity of confirming them. In addition, the study is limited to assessing simultaneously the two types of revascularization procedures (angioplasty and coronary revascularization) referring to the management of the antiplatelet agents, and it just does not assess the clinical impact of the antiplatelet therapy after the preoperative period. Therefore, we suggest that future studies address this prospective approach in order to size up the occurrence of thrombotic or hemorrhagic events during and after surgery.

## Conclusion

General surgeons stand for a group of physicians which follows the least the guidelines for managing antiplatelet agents in perioperative periods of non-cardiac surgeries. Divergences in conducts seem to stress the need of defining internal protocols, to divulge guidelines and continued education to ensure patients’ safety. Additionally, it was concluded that patients with more schooling, or a previous history of AMI, agree more with the cardiology guidelines, i.e., patients who have less schooling should be better accompanied in the management of the medicine therapy, and also to have more access to information about their health condition. However, fear of the possibility of a new infarction in a patient leads physicians no to hesitate to suspend the antiplatelet agent in non-cardiac surgical procedures when they are not neurosurgeries or transurethral resection of the prostate.
